# A long non-coding RNA signature to improve prognosis prediction of colorectal cancer

**DOI:** 10.18632/oncotarget.1895

**Published:** 2014-04-11

**Authors:** Ye Hu, Hao-Yan Chen, Chen-Yang Yu, Jie Xu, Ji-Lin Wang, Jin Qian, Xi Zhang, Jing-Yuan Fang

**Affiliations:** ^1^ Division of Gastroenterology and Hepatology, Ren Ji Hospital, School of Medicine, Shanghai Jiao Tong University, Shanghai Institution of Digestive Disease; Key Laboratory of Gastroenterology & Hepatology, Ministry of Health; State Key Laboratory of Oncogene and Related Genes., Shanghai, China; ^2^ Departments of Biochemistry and Molecular Biology, University of Texas MD Anderson Cancer Center, Houston, TX,USA

**Keywords:** colorectal cancer, lncRNAs, survival, GSEA

## Abstract

Increasing evidence suggests long non-coding RNAs (lncRNAs) are frequently aberrantly expressed in cancers, however, few related lncRNA signatures have been established for prediction of cancer prognosis. We aimed to develop a lncRNA signature to improve prognosis prediction of colorectal cancer (CRC). Using a lncRNA-mining approach, we performed lncRNA expression profiling in large CRC cohorts from Gene Expression Ominus (GEO), including GSE39582 test series(N=436), internal validation series (N=117); and two independent validation series GSE14333 (N=197) and GSE17536(N=145). We established a set of six lncRNAs that were significantly correlated with the disease free survival (DFS) in the test series. Based on this six-lncRNA signature, the test series patients could be classified into high-risk and low-risk subgroups with significantly different DFS (HR=2.670; P<0.0001). The prognostic value of this six-lncRNA signature was confirmed in the internal validation series and another two independent CRC sets. Gene set enrichment analysis (GSEA) analysis suggested that risk score positively correlated with several cancer metastasis related pathways. Functional experiments demonstrated three dysregulated lncRNAs, AK123657, BX648207 and BX649059 were required for efficient invasion and proliferation suppression in CRC cell lines. Our results might provide an efficient classification tool for clinical prognosis evaluation of CRC.

## INTRODUCTION

As the third leading culprit in cancer incidence worldwide [1], CRC continues to pose significant diagnostic, prognostic and therapeutic tribulations for clinicians. The American Joint Committee on Cancer (AJCC) TNM staging system is currently the only prognostic classification used in clinical practice to select patients for adjuvant chemotherapy [2-4]. However, the AJCC stage fails to predict recurrence accurately in many patients undergoing curative surgery for localized CRC. This highlights the need for new biomarkers for a more precise prediction of high-risk patients with CRC recurrence and consequently improved personalized cancer care.

Many studies have exploited microarray technology to investigate gene expression profiles (GEPs) in CRC in recent years, but only a small subset demonstrates clear prognostic significance [5-10]. Molecular markers such as mutations in Kirsten ras gene (KRAS) and BRAF as well as chromosome instability (CIN) and microsatellite instability (MSI) have been systematically analysed for prognostic potential in CRC[11]. So far, only KRAS mutation analysis has been used in clinical practice as a predictive marker for the effect of EGFR antibodies in metastatic disease [12-16]. Currently, the roles of dysregulated functional long non-coding RNAs (lncRNAs) in human cancers have received considerable attention [17-20]. LncRNAs are mRNA-like transcripts ranging in length from 200 nucleotides (nt) to~100 kilobases (kb) that lack significant protein-coding abilities [18, 21]. Increasing evidence suggests that these transcripts are frequently aberrantly expressed in cancers, and some of them have been implicated in diagnosis and prognostication [22, 23]. Searching a lncRNA signature might be of concrete predictive and prognostic value in the management of CRC.

Presently, a large group of lncRNA-specific probes were fortuitously represented on the commonly used microarray platform (Affymetrix HG-U133 plus 2.0), so we initially mined previously published gene expression microarray data from the Gene Expression Omnibus (GEO), and conducted lncRNA profiling on large cohorts of CRC patients. By using the sample-splitting method and Cox regression analysis, we identified a prognostic, six-lncRNA signature from the GSE39582 test series patients, and validated it in the internal validation series and another two independent GEO cohorts. To further confirm the reliability of the new signature we conducted a series of experiments to investigate three of six lncRNAs' biological behavior in CRC cell lines.

## RESULTS

### CRC data sets preparation

CRC data sets and corresponding clinical data were downloaded from the publicly available GEO databases. After removal of the samples without survival status, a total of 895 patients were analyzed, as detailed in Supplementary Table S1. These included 553 patients from GSE39582 (436 patients from the test series and 117 from the validation series), 197 patients from GSE14333 and 145 patients from GSE17536.

### Identification of prognostic lncRNAs from the test series

The 553 GSE39582 CRC patients were assigned to a test series (N=436) or a validation series (N=117). The test data set was used for the detection of prognostic lncRNAs. By subjecting the lncRNA expression data of the test series to univariable Cox proportional hazards regression analysis using the BRB-Array Tools, we identified a set of six lncRNAs that were significantly correlated with patients' DFS (P<0.0001; Table 1). Of these, a positive coefficient indicated that a higher expression level of two genes (AK024680 and AK026784) was associated with shorter survival. The negative coefficients for the remaining four genes (AK123657, CR622106, BX649059 and BX648207) indicated that their higher levels of expression were associated with longer survival.

**Table 1:** LncRNAs significantly associated with the disease free survival in the test series patients (N=436)

Gene symbol	Permutation P value^a^,^b^	Hazard ratio^a^	Coefficient
AK024680	5.47E-04	1.329	0.07826
AK123657	1.70E-05	0.713	−0.143
CR622106	1.23E-05	0.669	−0.19355
BX649059	5.71E-05	0.724	−0.00172
BX648207	1.46E-04	0.739	−0.20855
AK026784	1.99E-04	1.379	0.24326

a Derived from the univariable Cox proportional hazards regression analysis in the 436 test series patients.

b Obtained from permutation test repeated 10,000 times.

### The six-lncRNA signature and patients' survival in the test series

**Figure 1:**
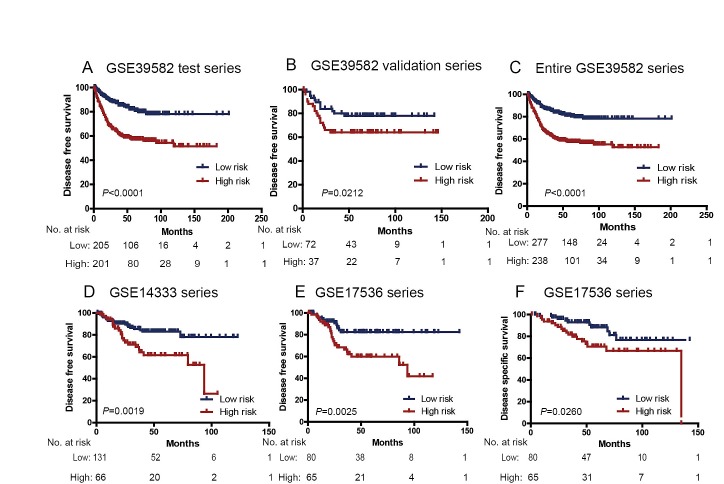
Kaplan–Meier estimates of the disease free survival (DFS) or disease specific survival (DSS) of GEO patients using the six-lncRNA signature The Kaplan–Meier plots were used to visualize the DFS probabilities for the low-risk versus high-risk group of patients based on the median risk score from the GSE39582 test series patients. (A) Kaplan Meier curves for GSE39582 test series patients (N=436); (B) Kaplan–Meier curves for GSE39582 validation series patients (N=117); (C) Kaplan–Meier curves for the entire GSE39582 patients (combined test and validation series patients, N=553). (D) Kaplan– Meier curves for GSE14333 patients (N=197); Kaplan–Meier curves for (E) DFS and (F) DSS of GSE17536 patients (N=145); The tick marks on the Kaplan–Meier curves represent the censored subjects. The differences between the two curves were determined by the two-sided log-rank test. The number of patients at risk was listed below the survival curves.

We created a risk-score formula according to the expression of these six lncRNAs for DFS prediction (see Material and methods), as follows: Risk score=(0.07826*expression level of AK024680)+(-0.14300* expression level of AK123657)+(-0.19355* expression level of CR622106)+(-0.00172* expression level of BX649059)+(-0.20855* expression level of BX648207)+(0.24326* expression level of AK026784). We then calculated the six-lncRNA signature risk score for each patient in the test series, and ranked them according to their risk score. As such, patients were divided into a high-risk group (N=218) or a low-risk group (N=218) using the median risk score of the test series as the cutoff point. Patients in the high-risk group had significantly shorter median DFS than those in the low-risk group (log-rank test *P*<0.0001) (Figure 1A). In details, the survival of patients in the low-risk group was 83.71% at 50 months, 78.09% at 100 months, and 78.09% at 150 months; which compared with 58.76%, 54.35% and 51.49%, respectively, in the high-risk group. The association of the six-lncRNA risk score and DFS was also significant when it was evaluated as a continuous variable in the univariable Cox regression model (Table 2).

**Table 2:** Univariable and multivariable Cox regression analysis in each data set

Variables	Univariable model^a^			Multivariable model^a^		
	HR	95% CI of HR	P value	HR	95% CI of HR	P value
GSE39582 test series(N=436)						
Six-lncRNA risk score	2.717	2.005 to 3.682	<0.0001	2.353	1.723 to 3.214	<0.0001
Age	1	0.988 to 1.012	0.9588	1.005	0.992 to 1.018	0.4308
AJCC stage						
1	1.00(referent)			1.00(referent)		
2	5.749	0.793 to 41.661	0.0835	4.051	0.557to 29.452	0.1669
3	9.263	1.284 to 66.828	0.0273	6.07	0.837 to 44.028	0.0745
4	31.434	4.281 to 230.822	0.0007	20.837	2.813 to 154.324	0.003
Gender	1.337	0.955 to 1.871	0.0911	1.585	1.126 to 2.232	0.0082
GSE39582 valiation series(N=117)						
Six-lncRNA risk score	2.271	1.424 to 3.622	0.0006	2.095	1.190 to 3.688	0.0103
Age	1.000	0.973 to 1.028	0.9815	1.02	0.987 to 1.054	0.2458
AJCC stage						
1	1.00(referent)			1.00(referent)		
2	2674096	0.000 to	0.9906	1994681	0.000 to	0.9916
3	9681475	0.000 to	0.9898	6897474	0.000 to	0.9909
4	1.57E+08	0.000 to	0.9880	1.52E+00	0.000 to	0.9891
Gender	1.060	0.543 to 2.073	0.8639	1.456	0.711 to 2.983	0.3044
Entire GSE39582 set(N=553)						
Six-lncRNA risk score	2.565	1.992 to 3.302	<0.0001	2.221	1.703 to 2.896	<0.0001
Age	1.000	0.989 to 1.011	0.9544	1.007	0.995 to 1.019	0.2574
AJCC stage						
1	1.00(referent)			1.00(referent)		
2	7.717	1.069 to 55.692	0.0427	5.203	0.718 to 37.701	0.1026
3	14.808	2.061 to 106.413	0.0074	9.431	1.305 to 68.148	0.0262
4	52.841	7.232 to 386.063	<0.0001	33.045	4.621to 250.808	0.0005
Gender	1.271	0.941 to 1.717	0.1177	1.534	1.130 to 2.083	0.0061
GSE14333 set(N=197)						
Six-lncRNA risk score	3.229	1.814 to 5.749	<0.0001	2.589	1.411 to 4.752	0.0021
Age	0.978	0.957 to 1	0.9544	0.991	0.968 to 1.015	0.4679
Duke stage^b^						
A	1.00(referent)			1.00(referent)		
B	2.927	0.665 to 12.889	0.1555	2.533	0.574 to 11.184	0.2200
C	6.897	1.639 to 29.025	0.0084	5.204	1.223 to 22.147	0.0256
Gender	1.118	0.612 to 2.042	0.7171	0.997	0.533 to 1.868	0.9935
GSE17536 set(N=145)						
Six-lncRNA risk score	3.25	1.658 to 6.368	0.0006	2.892	1.407 to 5.944	0.0039
Age	0.984	0.961 to 1.007	0.1595	0.996	0.970 to 1.022	0.7465
AJCC stage						
1	1.00(referent)			1.00(referent)		
2	5.382	0.695 to 41.708	0.1071	4.66	0.591 to 36.756	0.1422
3	10.645	1.428 to 79.327	0.021	8.445	1.109 to 64.275	0.0394
4	14.655	1.637 to 131.184	0.0164	14.329	1.572 to 130.581	0.0184
Gender	1	0.520 to 1.925	1	0.986	0.482 to 2.019	0.9703

Abbreviations: HR, hazard ratio; CI, confidence interval.

a In both univariable and multivariable Cox regression analyses, risk score and age were evaluated as continuous variables.

b In GSE14333 set, there was Duke stage instead of AJCC stage.

### Validation of the six-lncRNA signature for survival prediction in the validation series and the entire GSE39582 data set

**Figure 2:**
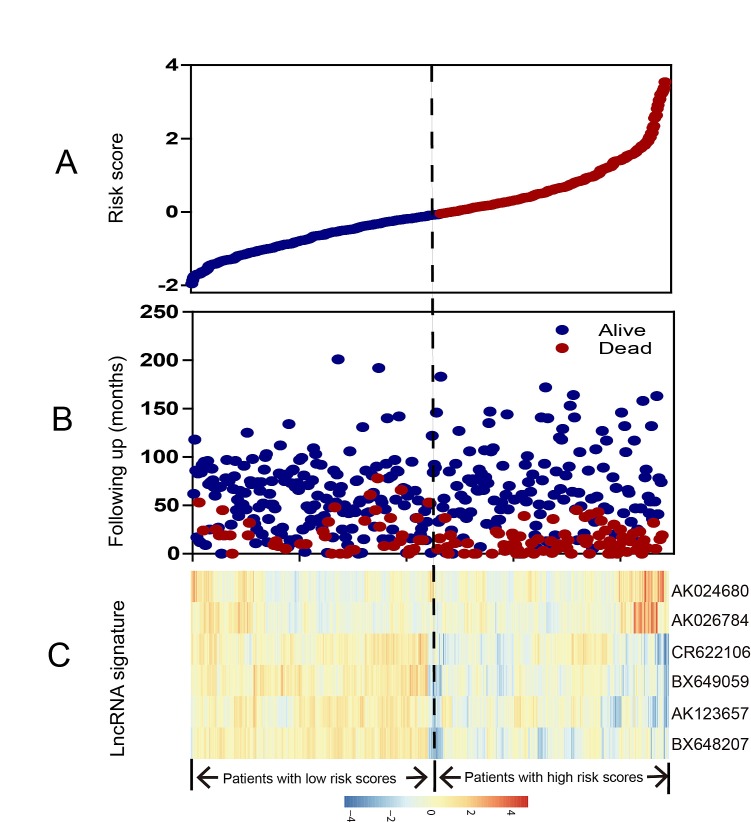
LncRNA risk score analysis of entire GSE39582 series The distribution of six-lncRNA risk score, patients' survival status and lncRNA expression signature were analyzed in the entire GSE39582 series patients (N=553). (A) LncRNA risk score distribution; (B) patients' survival status and time; (C) heatmap of the lncRNA expression profiles. Rows represent lncRNAs, and columns represent patients. The black dotted line represents the median lncRNA risk score cutoff dividing patients into low-risk and high-risk groups.

To confirm our findings, we validated our six-lncRNA signature in the internal validation series. By using the same risk score formula, we classified patients into a high-risk (N=44) and a low-risk group (N=73) using the median score of the test series as the cutoff point as for the validation series. In consistence with the findings described above, patients in the high-risk group had significantly shorter median DFS than those in the low-risk group (log-rank test *P*=0.0212) (Figure 1B). Risk score-based classification of the entire GSE39582 cohort (i.e. combined test and validation series) also yielded similar results (Figure 1C). The DFS for patients with low-risk scores was 82.15% at 50 months, 78.40% at 100 months, and 78.40% at 150 months; which compared with 58.80%, 55.17%, 52.77% in patients with high-risk scores, respectively. In the univariable Cox regression model that the six-lncRNA risk score was evaluated as a continuous variable, similar correlation could be observed (Table 2). We also showed the distribution of lncRNA risk score, the survival status of the CRC patients and the lncRNA expression signature. As shown in Figure 2, in the entire GSE39582 series patients, we found that patients with high-risk scores tended to express high levels of risky lncRNAs (AK024680 and AK026784) in their tumors, whereas patients with low-risk scores tended to express high levels of protective lncRNAs (the remaining four).

### Further validation of the six-lncRNA signature in another two independent data sets

We further validated our six-lncRNA signature in two independent CRC data sets obtained from GEO: GSE14333 and GSE17536. The clinical characteristics of these two cohorts are listed in Supplementary Table 1. These two datasets confirmed the ability of our model in predicting survival. As shown in Fig. 1D, the six-lncRNA model could effectively predict the DFS in patients from GSE14333 (log-rank test *P*=0.0019). For the GSE17536 set, patients could similarly be segregated into a high-risk group and a low-risk group (DFS: log-rank test *P*=0.0025 Figure 1E; DSS: log-rank test *P*=0.0260 Figure 1F). In the univariable Cox regression model, the lncRNA risk score was again significantly associated with DFS as a continuous variable in both GSE14333 and GSE17536 cohorts (Table 2).

### Prognostic value of the six-lncRNA signature is independent of AJCC stage and postoperative chemotherapy

**Figure 3:**
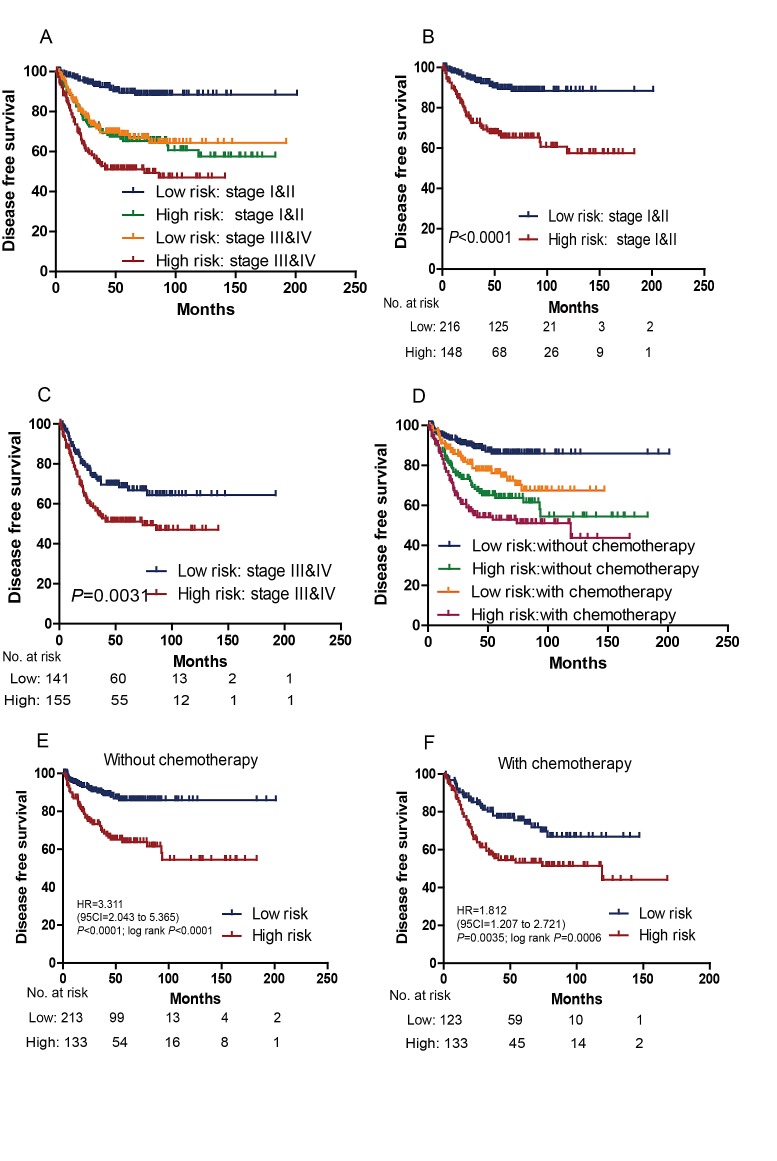
Kaplan–Meier estimates of the DFS of GSE39582 and GSE17536 patients with known AJCC stage (N=698) Patients were first stratified on the basis of AJCC stage (stage I and II vs stage III and IV) of their tumor samples. Kaplan–Meier plots were then used to visualize the survival probabilities for the low-risk versus high-risk group of patients based on the median risk score from the dataset patients within each stratum. (A) Kaplan–Meier curves for all patients (N=698); (B) Kaplan–Meier curves for stage I and II patients (N=371); (C) Kaplan–Meier curves for stage III and IV patients (N=327). The number of patients at risk was listed below the survival curves. Kaplan–Meier estimates of the DFS of GSE39582 and GSE14333 patients with known chemotherapy status. (D) Kaplan–Meier curves for all patients (N=622); (E) Kaplan–Meier curves for patients without chemotherapy (N=355); (F) Kaplan–Meier curves for patients with chemotherapy (N=267). The tick marks on the Kaplan–Meier curves represent the censored subjects. The differences between the two curves were determined by the two-sided log-rank test.

We tested whether the prognostic value of the six-lncRNA signature was independent of AJCC stage. For this, we first performed multivariable Cox regression analysis that included lncRNA risk score, age, and other clinical characteristics such as gender and AJCC stage (when available) as covariables. The results showed that the six-lncRNA risk score remained to be significantly associated with DFS when adjusted by AJCC stage and other variables in every cohort (Table 2). Data stratification analysis was then performed, which stratified the two GEO (GSE39582 and GSE17536) patients (N=698) into a stage I and II stratum (without lymph nodes metastasis) or a stage III and IV stratum (with lymph nodes metastasis). This analysis showed that within each stage stratum, the six-gene risk score could further subdivide the patients into those likely to have longer survival and those likely to have shorter survival (Figure 3A-C), in a manner similar to what we observed in the entire group of patients, results of separate series see Supplementary Figure S1.

Prognostic value of the signature for the patients with or without postoperative chemotherapy was also assessed. In the combined GSE39582 and GSE14333 series (when chemotherapy information available), high-risk score significantly correlated with an unfavourable DFS in those with and without postoperative chemotherapy (Figure 3D-F), results of separate series see Supplementary Figure S1& Figure S2.

### Identification of six-lncRNA signature associated biological pathways and processes

**Figure 4:**
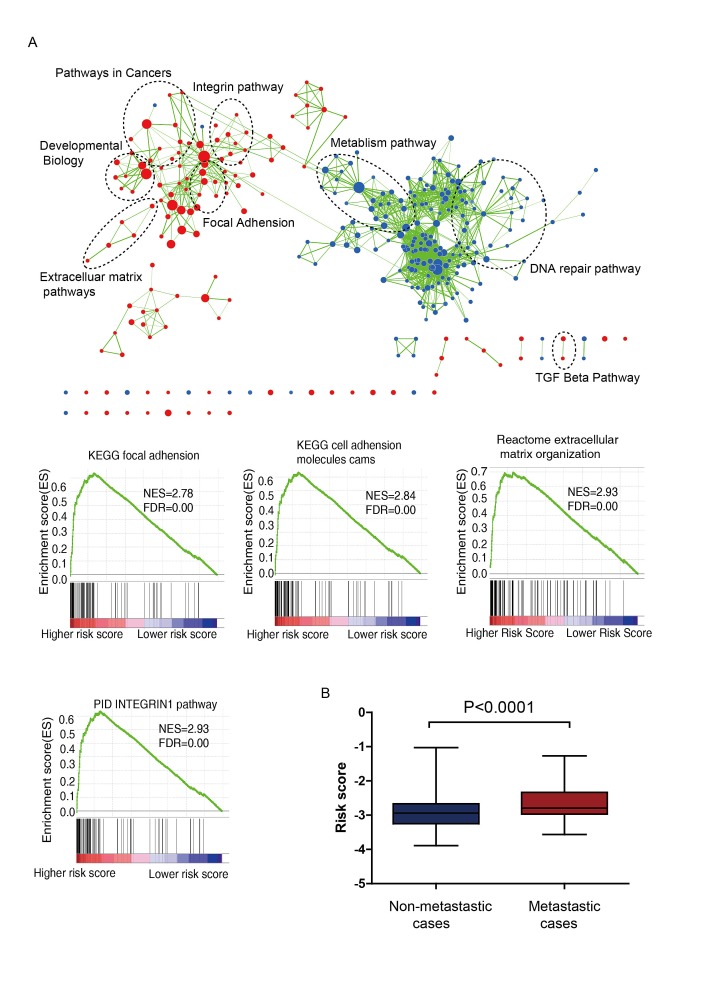
(A) Gene Set Enrichment Analysis Delineates Biological Pathways and Processes associated with risk score Cytoscape and Enrichment Map were used for visualization of the GSEA results. Nodes represent enriched gene sets, which are grouped and annotated by their similarity according to related gene sets. Enrichment results were mapped as a network of gene sets (nodes). Node size is proportional to the total number of genes within each gene set. Proportion of shared genes between gene sets is represented as the thickness of the green line between nodes. (B) Risk score of patients with or without distant metastasis in combined GSE39582 and GSE17536 data (N=698, *P*=0.0001).

We performed GSEA to identify associated biological processes and signaling pathways using the lncRNA signature based risk score for classification. Significant gene sets (FDR < 0.01, p <0.005) were visualized as interaction networks with Cytoscape and Enrichment Map (Figure 4a, Supplementary Table S2). The risk score was accompanied with up-regulation of several cancer-related networks, namely Integrin pathway, Extracelluar matrix pathways, Focal adhesion. These related pathways were reported to affect cancer metastasis [24-26], and thus the signature might be involved with. Since cancer metastasis is a major influential factor for DFS, we compared the risk score of patients with and without distant metastasis in GSE39582 and GSE17536 series (when the information of AJCC stage available), for which the AJCC stage IV stands for distant metastasis, otherwise no distant metastasis occurs. Surprisingly, patients with distant metastasis tended to get higher risk score than patients without distant metastasis (Figure 4b, *P*<0.0001), results of separate series see Supplementary Figure S3&Figure S4.

### Three signature lncRNAs regulate the proliferation and invasion abililty of CRC cell lines

**Figure 5:**
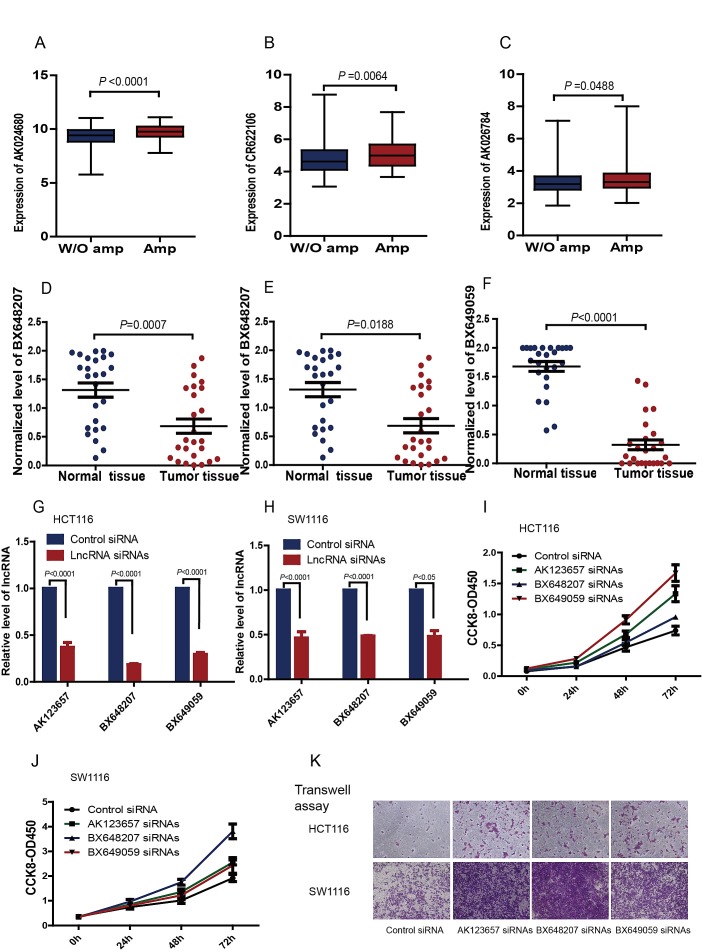
Box plot of (A) AK024680, (B) CR622106 and (C) AK026784 and expression in CRC with genomic amplification (Amp; N=76, N=73 and N=237, respectively) and without genomic amplification (W/o amp; N=326, N=329 and N=165, respectively) MWU test was used to determine the significance of the comparisons. Expression of (D) AK123657, (E) BX648207 and (F) BX649059 were quantified by qRT-PCR in 25 pairs of CRC tissues and their matched normal tissues. AK123657 (*P*=0.0007, Paired ttest), BX648207 (*P*=0.0188, Paired ttest) and BX649059 (*P*<0.0001, Paired ttest) expression were significantly decreased in CRC tissues compared with their normal tissues. To evaluate the effects of AK123657, BX648207 and BX649059 on cell biological behaviors, specific small interfering RNAs (siRNAs) were employed to knockdown the lncRNAs expression in human CRC HCT116 (G) and SW1116 (H) cells. Cell-counting kit-8 assays indicated that cell proliferation was increased when AK123657, BX648207 and BX649059 were knockdown (I & J). We further used Transwell assay to monitor the effect of manipulating AK123657, BX648207 and BX649059 expression on cell invasiveness. Knockdown of AK123657, BX648207 and BX649059 significantly increase the number of HCT116 and SW1116 cells that penetrated the Transwell filter (K), which suggested a substantial gain of cell invasion ability.

The analyses of CGH arrays revealed that CRC samples shared a typical DNA copy alteration pattern (Supplementary Figure S5). The signature lncRNAs AK024680, CR622106 and AK026784 showed positive correlations between their somatic copy-number alterations (SCNAs) and expression levels (Figure 5 A-C). While, for AK123657, BX648207 and BX649059, the DNA copy alterations information was unavailable, thus we selected AK123657, BX648207 and BX649059 among the six lncRNAs for further experimental validation and analyzed their expression in 25 pairs of CRC and corresponding nontumor colorectal tissues, using quantitative real-time polymerase chain reaction (qRT-PCR). These data confirmed that AK123657 (*P*<0.0001), BX648207 (*P*=0.0188) and BX649059 (*P*<0.0001) expression were decreased in CRC cancer (Figure 5D-F).

To evaluate the effects of AK123657, BX648207 and BX649059 on cell biological behaviors, specific small interfering RNAs (siRNAs) were employed to knockdown AK123657, BX648207 and BX649059 expression in human CRC cell lines, HCT116 and SW1116. In case of target-off effect, two small interfering RNAs were used for each lncRNA (Figure 5G&Figure 5H). Cell-counting kit-8 assays indicated that cell proliferation was increased when AK123657, BX648207 and BX649059 was knocked down (Figure 5I&Figure 5J).These results suggested that AK123657, BX648207 and BX649059 played a physiological role in regulating cell proliferation. We further used Transwell assay to monitor the effect of manipulating AK123657, BX648207 and BX649059 expression on cell invasiveness. Knockdown of these lncRNAs significantly increased the number of CRC cells that penetrated the Transwell filter, which demonstrated a substantial gain of cell invasion ability (Figure 5K).

## DISCUSSION

The conventional view of gene regulation in biology has centered around protein-coding genes until the discovery of thousands of lncRNAs. Numerous reports of dysregulated lncRNA expression across numerous cancer types suggest that abnormal lncRNA expression may be a major contributor to tumorigenesis. The aberrant expressions of specific lncRNAs in cancer could mark the spectrum of disease progression and these lnRNAs may serve as independent biomarkers for diagnosis and prognosis [27, 28]. More recently, lncRNAs have been implicated in CRC pathogenesis [29, 30]. However, the prognostic values of lncRNAs in CRC have not yet been investigated. To explore the prognostic lncRNA genes, we profiled lncRNA by mining the existing microarray gene expression data since a great number of lncRNAs were interrogated on many commonly used commercial arrays [27, 28]. By analyzing the associations between lncRNA expression profiles and clinical outcome of CRC patients in GEO date set, we identified a six-lncRNA signature that was significantly associated with the DFS. Base on our knowledge, this is the first report that relates lncRNA expression patterns with patients' prognosis in CRC.

By applying the six-lncRNA signature to the GSE39582 test series patients, a clear separation was observed in the survival curves between patients with low- or high-risk signatures. Patients with a low-risk six-lncRNA signature in their tumor specimens tended to have prolonged overall survival, whereas patients with a high-risk signature tended to have shortened survival. The association between the lncRNA signature and survival was significant regardless of the former was evaluated as a continuous variable or category variable (dividing by the median cutoff). The usefulness of this lncRNA signature could be internally validated in the non-overlapping GSE39582 patients (the validation series) and two independent cohorts of GSE14333 and GSE17536 that were profiled through the same platform of GSE39582 patients, indicating good reproducibility of this lncRNA signature in CRC.

Further analysis revealed that the prognostic value of the six-lncRNA signature was independent of one of the main prognostic factors-AJCC stage. Currently, AJCC stage has been widely accepted as a powerful predictor of treatment response and survival in CRC. In general, stage I and II shows better survival, whereas stage III and IV is correlated with worse survival in a treatment-independent manner. In accordance with other studies, AJCC stage was a significant prognostic factor in our study when assessed in the univariable Cox regression analysis (Table 2). Therefore, it will be important to evaluate whether the prognostic value observed on our six-lncRNA signature is independent of this known strong prognostic factor or not. Here, by performing multivariable Cox regression analysis and stage stratification analysis, we showed the stage-independent prognostic values of the six-lncRNA signature in CRC patients. The exception was GSE14333, in which we did not have the AJCC stage information. Nonetheless, combining all these results together, we could conclude that the prognostic value of the lncRNA signature observed in our study was independent of AJCC stage. Moreover, it was of interest to find that the six-lncRNA signature had a similar survival predictive ability as AJCC stage in the receiver operating characteristic (ROC) analysis. Six-lncRNA signature combine AJCC stage has a stronger power for DFS prediction in the ROC analysis (Supplementary Figure S6). The ability of our six-lncRNA signature in identifying subgroups of CRC patients with identical AJCC stage implies that lncRNA signatures may be used to refining the current prognostic model and facilitating further stratification of patients in the future clinical trials.

Our signature predicted an unfavourable prognosis of those with or without postoperative chemotherapy in combined cohorts, indicating that some signature lncRNAs might involve in chemotherapy response and contribute to therapeutic outcome, thus needed to be studied further.

Examination of associated molecular pathways revealed that the six-lncRNA signature was more likely to involve with Integrin pathway, extracelluar matrix pathways and focal adhesion. Extracellular matrix via its receptors, the integrins, and kinases downstream of β1 integri-focal adhesion kinase (FAK) have emerged as a major pathway leading to several cellular responses including migration, differentiation, and proliferation [31, 32]. Furthermore, patients with distant metastasis were validated in our study to achieve higher risk score than patients without distant metastasis. Studied have revealed that distant metastasis played a significant role in DFS, cases without distant metastasis were more likely to get prolonged DFS and vice versa [33, 34]. Therefore, the significant signaling pathways might support that lncRNA signature has DFS prediction power and suggest possible avenues for future targeted therapies.

However, gene signatures attempted to serve as prognostic and/or predictive markers for CRC hardly achieve widespread use despite accumulating signatures have been explored [6-9, 35]. This may partly result from ignoring of biologic rationale during gene selection, which may derive from computer-based algorithms that incorporate a cluster of unrelated genes. SCNAs is an important form of somatic genetic alteration in cancer, and within that a genomic region is either amplified or deleted. Some of the genes within amplified (or deleted) regions exhibit increased (or decreased) expression levels resulting in altered activity in cancer cells. As lncRNAs do not have proteins encoding abilities, their functions are closely related with their transcript abundance. The signature lncRNAs AK024680, CR622106 and AK026784 showed positive correlations between their SCNAs and expression levels, which we reasoned could possibly lead to altered lncRNA activity in CRC. The other three lncRNAs from the gene signature were functional studied by further experiments. We focused on the lncRNAs' biological function and analyzed their relation with clinicopathological features. AK123657, BX649059 and BX648207 were significantly down-regulated in CRC tissues compare to normal colorectal tissues, suggesting a protective role in CRC biogenesis. Cell proliferation and invasion ability were enhanced after decreasing expression of these lncRNAs, which was in accordance with the gene signature that patients suffered distant metastasis achieve higher risk score than patients without distant metastasis.

The limitations should be acknowledged for this study. In particular, treatment response was not included because this information was not available for a substantial proportion of cases. Thus, the significance and robustness of the signature as a prognostic classification requires further confirmation, ideally with large prospective patient cohorts included in adjuvant trials.

The present study demonstrated the associations between the expressions of these lncRNAs and DFS of CRC patients. The findings may identify high-risk patients for more intensive adjuvant therapy in addition to the standard regimen; low-risk patients, on the other hand, may not need to receive intensive, and potentially toxic, therapies.

Biological behavior of three lncRNAs from the gene signature was studied because we supposed gene signature consisted of functional lncRNAs tended to have better prediction. However, the roles of the other lncRNAs in CRC pathogenesis are presently unclear, and our findings suggest that they deserve further studied. Additional functional investigations of these lncRNAs on cancer cell lines and xenograft models may increase our outstanding of their roles in determining CRC prognosis. Last but not least, all the lncRNAs were derived through the re-annotation algorithm in this study and should be validated in the further studies.

In conclusion, this study presents a powerful lncRNA signature by probing and integrating currently available microarray data. This innovative lncRNA signature showed independence of one of the main prognostic factors-AJCC stage and the signature might help personalize prediction of CRC prognosis. The GSEA analysis suggested that the signature might involve with several cancer pathways, most likely with cancer metastasis, which provided support for DFS predictive ability of the signature. These identified signature lncRNAs played vital roles in the CRC cell lines, which might serve as alternative biomarkers and therapeutic targets for CRC.

## MATERIALS AND METHODS

### Microarray data processing and lncRNA profile mining

The raw CEL files were downloaded from GEO database and background were adjusted using Robust Multichip Average. GATExplorer was used to process microarrays on a local computer for gene expressions of lncRNAs [36]. This GATExplorer provides a series of R packages, designed to be used with BioConductor tools, that allow to apply in a simple way the probe mapping data included in GATExplorer. A type of files called ncRNA Mapper were also obtained from GATExplorer, which include the probes that do not map to any coding region but that were mapped to a database for non-coding RNA of human and mouse derived from RNAdb [37]. A customized R scripts was used to perform a microarray expression calculation according to the re-mapping data(file ncrnamapperhgu133plus2cdf_3.0). Each LncRNA should include at least a minimum of 3 probes mapping in the corresponding ncRNAs entity.

### GSEA

GSEA was performed by the JAVA program (http://www.broadinstitute.org/gsea) using MSigDB C2 CP: Canonical pathways gene set collection(1320 gene sets available).The GSEA, visualized in Cytoscape (version 2.8.2), and the Enrichment Map software[38], was used to determine if the members of a given gene set were generally associated with risk score, and was therefore performed on all mRNA genes on the HG-U133 Plus 2.0 ranked by enrichment score from most negative to most positive. 1000 random sample permutations were carried out, and the significance threshold set at FDR<0.01. If a gene set had a positive enrichment score, the majority of its members had higher expression accompanied with higher risk score, and the set was termed “enriched”.

### Statistical analysis

The association between the lncRNA expression and patient's disease free survival (DFS) and disease specific survival (DSS) was assessed by univariable Cox regression analysis along with a permutation test using Biometric Research Branch-Array (BRB-Array) Tools [39]. Genes were considered statistically significant if their permutation *P* values were less than or equal to 0.01. To construct a predictive model, the selected genes were fitted in a multivariable Cox regression model in the test series as described. A risk score formula was then established by including each of these selected genes, weighted by their estimated regression coefficients in the multivariable Cox regression analysis. With this risk score formula, patients in each set were classified into high-risk or low-risk group by using the median risk score of the test series as the cutoff point. Survival differences between the low-risk and high-risk groups in each set were assessed by the Kaplan–Meier estimate, and compared using the log-rank test. To test whether the risk score was independent of AJCC stage, multivariable Cox regression analysis and data stratification analysis were performed. We used ROC curves to compare the sensitivity and specificity of the survival prediction based on the lncRNA risk score and AJCC stage. In the log-rank test, Cox regression analysis and ROC analysis, the significance was defined as *P* values being less than 0.05.

A total of 402 of the 553 primary CRC samples from the entire GSE39582 cohort could be analyzed for array-based comparative genomic hybridization (CGH). To exam whether lncRNAs within amplified (or deleted) regions have increased (or decreased) expression levels, Mann-Whitney *U* (MWU) test was used to determine the significance of the comparisons.

### Cell viability assays

Cell viability was assessed by the Cell Counting Kit 8 (CCK-8; Dojindo) as described previously [40, 41]. Briefly, control and treated HCT116 and SW1116 cell lines were seeded into 96-well plates at an initial density of 5000cells/well. At each time points, 10 μl of CCK-8 solution was added to each well and incubated for 2 h. The absorbance was measured by scanning with a microplate reader at 450 nm. Data were expressed as the as follows: relative viability= A450 (treated) − A450 (blank) or (A450 (control) − A450 (blank).

### Sequences of siRNAs

AK123657 siRNA1 CCUCCAGACUGUGAGUAAUTT AUUACUCACAGUCUGGAGGTT

siRNA2 GGAGGUGCAUGACUAACAATT UUGUUAGUCAUGCACCUCCTT

BX648207 siRNA1 GGCCUGAAUUUGGUUACAUTT AUGUAACCAAAUUCAGGCCTT

siRNA2 GCCACUUGCAAGUGGAAUATT UAUUCCACUUGCAAGUGGCTT

BX649059 siRNA1 GGCUUAAAGUAGGUAUUUATT UAAAUACCUACUUUAAGCCTT

siRNA2 CCUGAAGAGUACAGAUAAATT UUUAUCUGUACUCUUCAGGTT

### Tumor cell invasion assays

Tumor cell invasion assays were performed using Boyden chambers with filter inserts (pore size, 8-μm, Millipore) coated with Matrigel (40μm; BD Biosciences) in 24-well dishes (Corning) as described previously [42]. Briefly, 3×10^5^ cells after transfected with lncRNA siRNAs or control siRNA were seeded in the upper chamber, while the RPMI-1640 medium (Invitrogen) supplemented with 30% fetal bovine serum was placed in the lower chamber. The plates were incubated for 48h. Then the cells were fixed in 4% formaldehyde and stained with 0.05% crystal violet for 20min at room temperature. Cells on the upper side of the filters were removed by cotton-tipped swabs, and the filters were washed with PBS. The cells on the lower side of the filters were defined as invasive cells.

## SUPPLEMENTARY FIGURES AND TABLES




