# Supplementary material for: A long non-coding RNA signature to improve prognosis prediction of colorectal cancer
Source: Oncotarget. 2014 Apr 11;5(8):2230–42. doi: 10.18632/oncotarget.1895 (PMC4039159; doi:10.18632/oncotarget.1895)
Supplement: Supplementary file 1 [file oncotarget-05-2230-s001.pdf]

A long non-coding RNA signature to improve prognosis prediction of colorectal cancer

Supplementary information

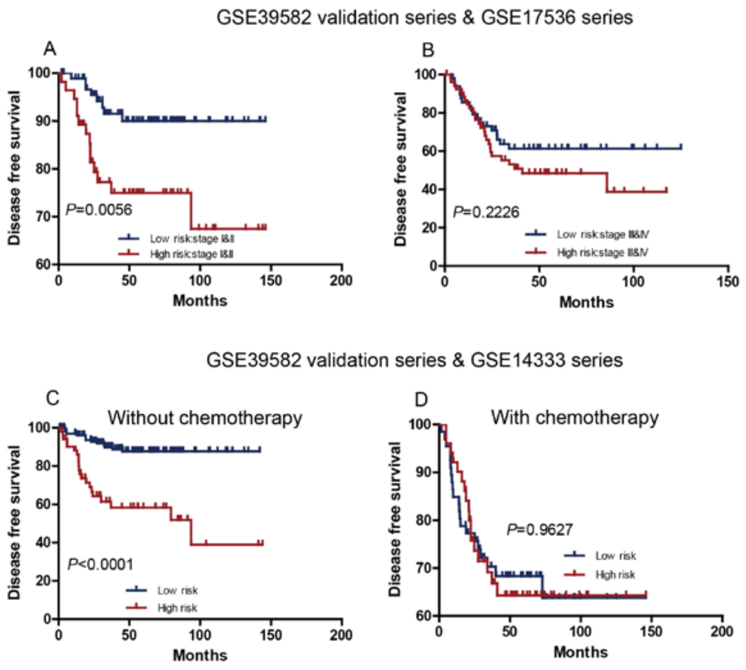

Supplementary Figure S1: (A-B) Kaplan–Meier estimates of the DFS of combined GSE39582 validation and GSE17536 series patients with known AJCC stage information. (C-D) Kaplan–Meier estimates of the DFS of combined GSE39582 validation and GSE14333 series patients with known chemotherapy status

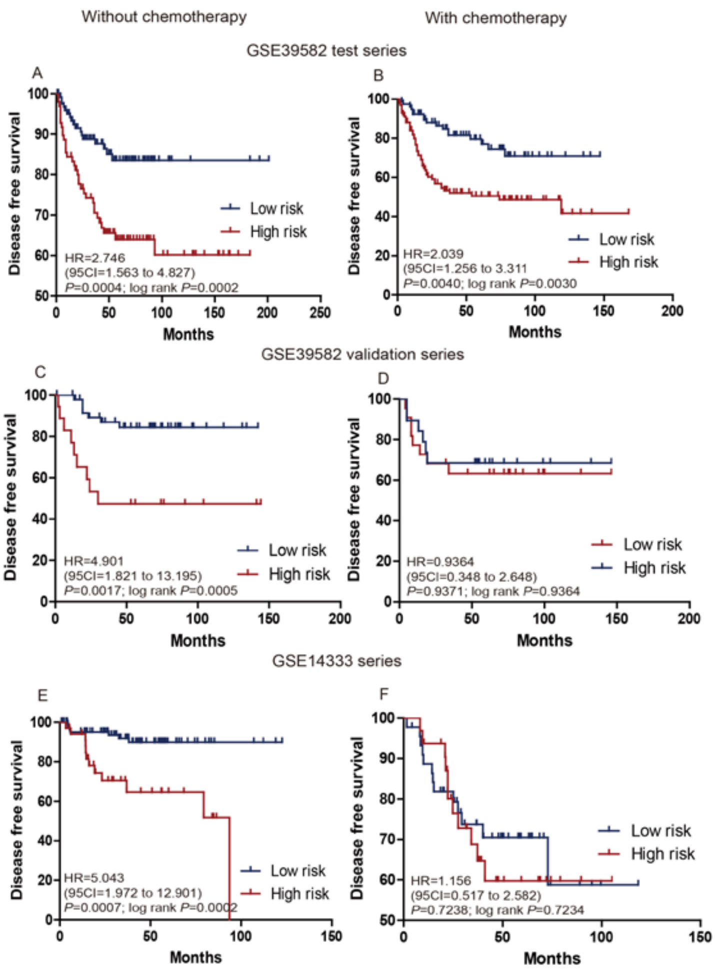

Supplementary Figure S2: Kaplan–Meier estimates of the DFS of GSE39582 and GSE14333 patients with known chemotherapy status (A-B) GSE39582 test series; (C-D) GSE39582 validation series; and (E-F) GSE14333 series.

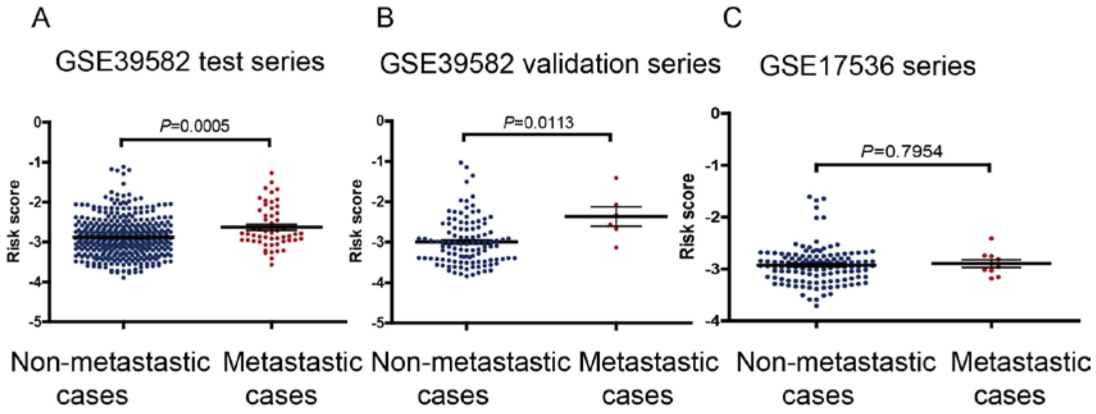

Supplementary Figure S3: Risk score of patients with or without distant metastasis in (A)GSE39582 test series; (B) GSE39582 validation series; and (C) GSE17536 series.

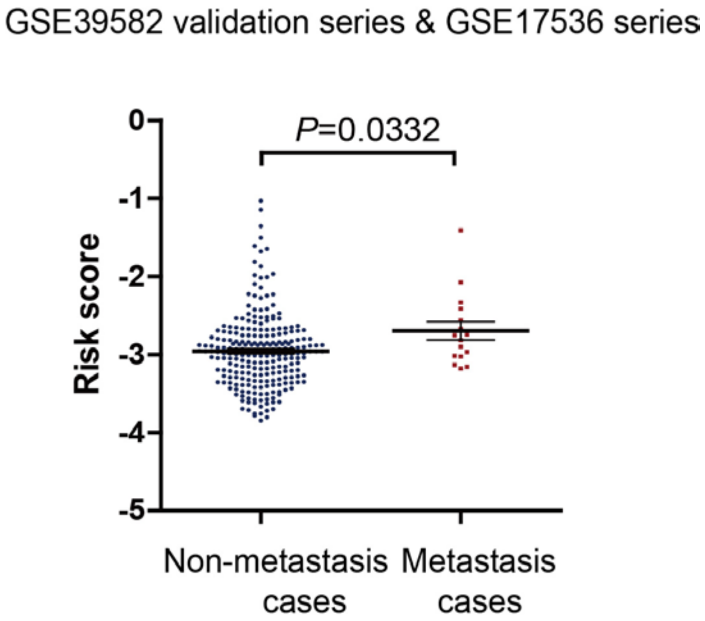

Supplementary Figure S4: Risk score of patients with or without distant metastasis in combined GSE39582 validation and GSE17536 series.

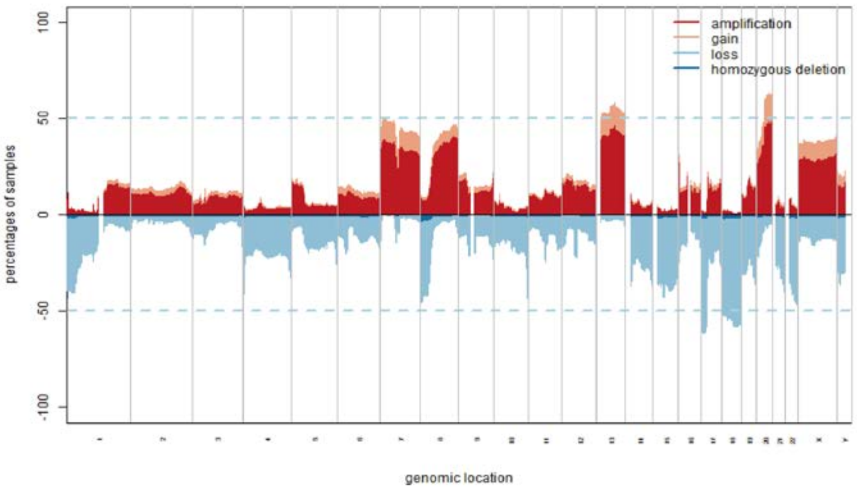

Supplementary Figure S5: Frequencies of gain and loss across 402 arrays with available CGH data of GSE39582(GSE40966).

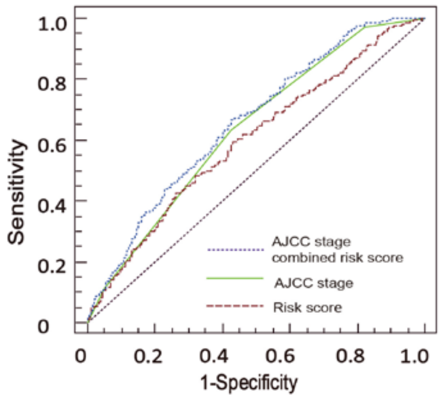

	AUC (95% CI)	*P* value
AJCC stage combined risk score	0.658 (0.622-0.694)	
AJCC stage	0.599 (0.561-0.635)	0.0015
Risk score	0.636 (0.599-0.672)	0.0215

Supplementary Figure S6: ROC analysis of the sensitivity and specificity of the disease free survival prediction by the six-lncRNA risk score, AJCC stage in GSE39582 and GSE17536 patients with known AJCC stage (n = 698). *P* values were from the comparisons of the area under the ROC (AUROC) of six-lncRNA risk score versus those of AJCC stage and six-lncRNA risk score combine with AJCC stage, respectively. As can be seen, the six-lncRNA risk score combine with AJCC stage showed a better prediction of DFS than risk score (*P* = 0.0015), and AJCC stage(*P* = 0.0215) alone.
